# Manipulating Fano Coupling in an Opto‐Thermoelectric Field

**DOI:** 10.1002/advs.202412454

**Published:** 2025-01-21

**Authors:** Linhan Lin, Sergey Lepeshov, Alex Krasnok, Yu Huang, Taizhi Jiang, Xiaolei Peng, Brian A. Korgel, Andrea Alù, Yuebing Zheng

**Affiliations:** ^1^ State Key Laboratory of Precision Measurement Technology and Instruments Department of Precision Instrument Tsinghua University Beijing 100084 P. R. China; ^2^ Department of Electrical and Photonics Engineering DTU Electro Technical University of Denmark Building 343 Lyngby DK‐2800 Kgs Denmark; ^3^ Department of Electrical and Computer Engineering Florida International University Miami Florida 33174 USA; ^4^ School of Physics and Electronics Hunan University Changsha 410082 P. R. China; ^5^ Mc Ketta Department of Chemical Engineering The University of Texas at Austin Austin TX 78712 USA; ^6^ Materials Science & Engineering Program and Texas Materials Institute The University of Texas at Austin Austin TX 78712 USA; ^7^ Department of Electrical and Computer Engineering The University of Texas at Austin Austin TX 78712 USA; ^8^ Photonics Initiative Advanced Science Research Center City University of New York New York NY 10031 USA; ^9^ Physics Program Graduate Center City University of New York New York NY 10016 USA; ^10^ Walker Department of Mechanical Engineering The University of Texas at Austin Austin TX 78712 USA

**Keywords:** Fano resonance, Mie scattering, nanoparticle assembly, optical trapping, opto‐thermoelectric manipulation

## Abstract

Fano resonances in photonics arise from the coupling and interference between two resonant modes in structures with broken symmetry. They feature an uneven and narrow and tunable lineshape and are ideally suited for optical spectroscopy. Many Fano resonance structures have been suggested in nanophotonics over the last ten years, but reconfigurability and tailored design remain challenging. Herein, an all‐optical “pick‐and‐place” approach aimed at assembling Fano metamolecules of various geometries and compositions in a reconfigurable manner is proposed. Their coupling behavior by in situ dark‐field scattering spectroscopy is studied. Driven by a light‐directed opto‐thermoelectric field, silicon nanoparticles with high‐quality‐factor Mie resonances (discrete states) and low‐loss BaTiO_3_ nanoparticles (continuum states) are assembled into all‐dielectric heterodimers, where distinct Fano resonances are observed. The Fano parameter can be adjusted by changing the resonant frequency of the discrete states or the light polarization. Tunable coupling strength and multiple Fano resonances by altering the number of continuum states and discrete states in dielectric heterooligomers are also shown. This work offers a general design rule for Fano resonance and an all‐optical platform for controlling Fano coupling on demand.

## Graphical abstract

Meta‐atoms of barium titanate nanoparticles and silicon nanoparticles are treated as continuum states and discrete states, respectively, and assembled into Fano metamolecules in an opto‐thermoelectric field. The Fano resonances are manipulated on‐demand by tuning the resonant frequency of the discrete states, the number of continuum states or discrete states, or the polarization of incident light.

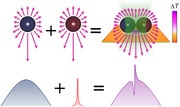

## 1 Introduction

Fano resonances, which were first explained by Ugo Fano as the interference between a discrete state and a continuum state in a quantum mechanical system, feature an asymmetric line shape in the absorption spectra of noble gases.^[^ ^1^ ^]^ Fano resonances have been observed in a variety of quantum systems, such as quantum dots and nanowires.^[^ ^2^ ^]^ This unusual interference phenomenon is not just found in quantum systems, it is also common in optics and photonics.^[^ ^3^, ^6^ ^]^ In nanophotonics, Fano resonances are associated with resonant optical phenomena, such as surface plasmons and Mie resonance, featuring a sharp transition at an extremely narrow frequency window in the optical spectroscopy (such as transmission, scattering, or absorption).^[^ ^7,^, ^21^ ^]^ Narrow Fano resonances have been harnessed to design different high‐performance optical devices in optical sensing,^[^ ^22^, ^23^ ^]^ nonlinearity,^[^ ^24,^, ^26^ ^]^ optical chirality,^[^ ^27^ ^]^ optical display,^[^ ^28^ ^]^ and more.

Fano resonances have been observed in various photonic nanostructures, including individual asymmetric nanostructures,^[^ ^9^, ^29^ ^]^ nanoparticle assemblies,^[^ ^7^, ^11^, ^12^, ^14^, ^18^, ^30^ ^]^ metamaterials,^[^ ^25^, ^27^, ^31^, ^32^ ^]^ and photonic crystals.^[^ ^33,^, ^35^ ^]^ In optics, the key to achieving Fano resonances is to combine a spectrally narrow mode with a much broader one. In homogenous clusters of nanoparticles, since each individual particle has a similar optical response, the generation of discrete states and continuum states relies on the mode hybridization, which complicates the structural design. A typical example is plasmonic nanoclusters consisting of packed metallic nanoscale components, i.e., a central particle surrounded by six equivalent particles.^[^ ^36^ ^]^ The basic design rule is to obtain an equivalent dipole moment of the surrounding ring structure with the central particle, leading to a subradiant antibonding state coupled with a superradiant bonding state. The spectral feature of Fano resonance in these plasmonic clusters can be carefully designed by varying the composition, particle size, interparticle gap, and geometric arrangement.^[^ ^36^, ^38^ ^]^ Optical sensing and advanced nanolasers that rely on Fano resonances require precise control over the Fano resonance shape and spectral position.^[^ ^39^, ^40^ ^]^ However, real‐time control of Fano resonances and spectral lineshapes is still challenging.

From another perspective, the two modes in Fano interference can be created from individual components in the nanoclusters. Specifically, optical nanocavities made of different materials can be explored to control Fano interference. It has been demonstrated that electrostatically assembled Au‐Ag nanodimers exhibit Fano resonance in the absorption spectra, indicating the possibility of making photonic Fano nanostructures through bottom‐up assembly.^[^ ^16^ ^]^ The nanoparticles were labeled with different surface charges, allowing selective interaction during the assembly. However, the variety and spread of the nanoparticles complicate the precise control of geometry and optical response of the nanocluster. It is still an appealing perspective to selectively pick up nanoparticles with different optical properties and assemble them into photonic nanoclusters at will to investigate the coupling behavior.

**Figure 1:**
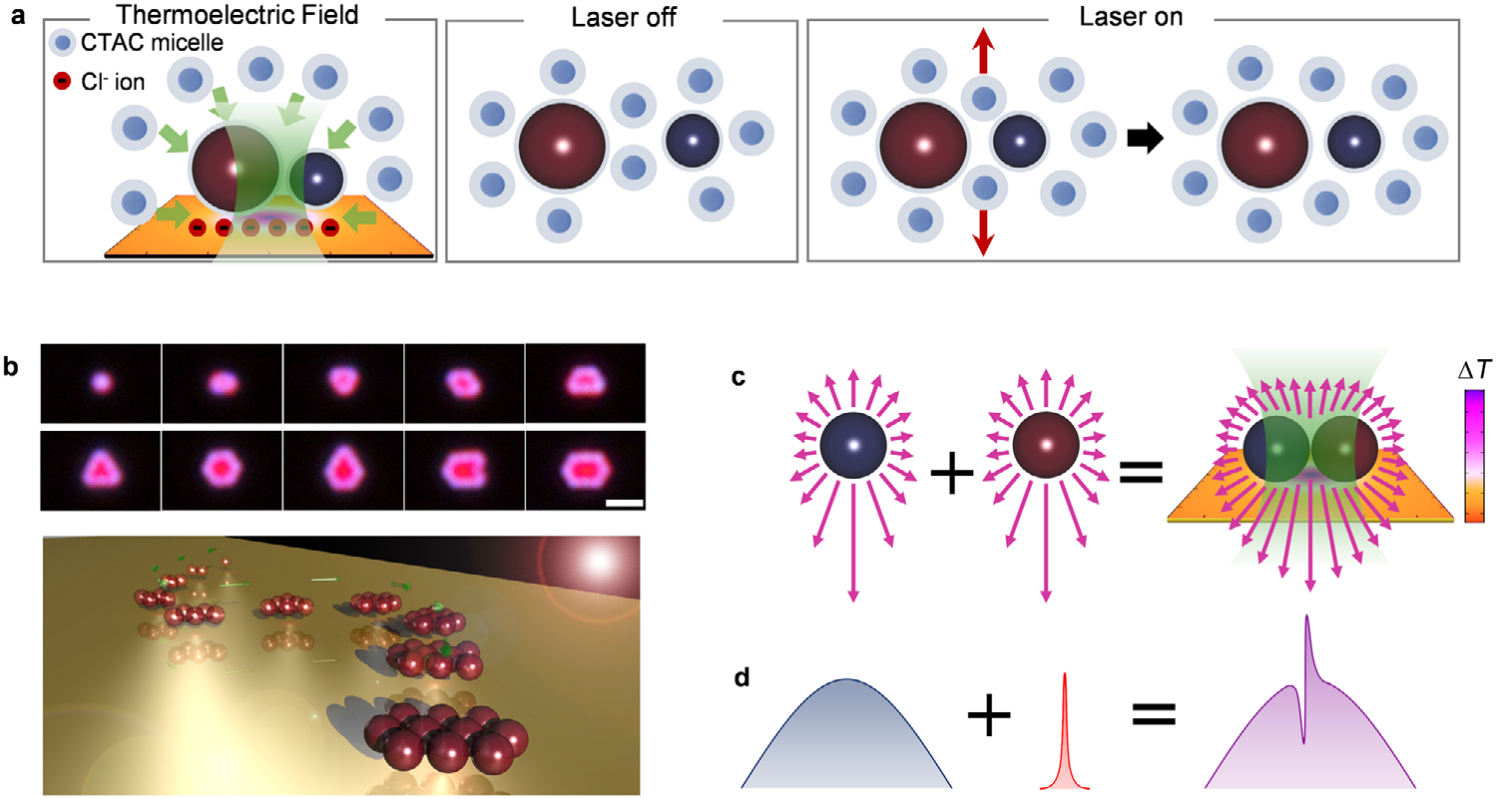
Design concept and working principle. A, Schematics of the thermoelectric field induced assembling between BaTiO3 and Si nanoparticles under laser irradiation. When laser is on, thermophoresis drives spatial separation between CTAC micelles and Cl^−^ ions through the different Soret coefficients and creates an opto‐thermoelectric field pointing from hot to cold regions. Driven by the opto‐thermoelectric field, the CTAC‐adsorbed BaTiO3 and Si nanoparticles are captured and assembled at the laser spot (hot region). The green arrows represent the direction of the opto‐thermoelectric field, which points to the laser spot. The red arrows indicate the depletion of CTAC micelles. b, Scheme (bottom) and dark‐field optical images (top) showing the successive assembly of 500 nm SiNPs into diverse oligomers. Scale bar: 2 µm. c and d, the Design concept of the Fano heterodimer. Two individual particles, one exhibiting a broad scattering spectrum while another exhibiting a narrow scattering spectrum, are assembled into a heterodimer using a light‐controlled temperature field. The arrows represent the scattering signal of the particles.

To address this challenge, we propose an all‐optical approach to “pick‐and‐place” different nanoparticles into hetero‐metamolecules, with their coupling behavior investigated using in situ spectroscopy. The principles of opto‐thermoelectric assembly are sketched in **Figure** **1**a. Technically, a focused laser beam is used to heat a thin Au film, creating a thermal hot spot due to optical heating. Cetyltrimethylammonium chloride (CTAC) surfactant was added into the nanoparticle suspension, providing the macro cations (CTAC micelles) and counter Cl^−^ ions. Since CTAC micelles and Cl^−^ ions have different Soret coefficients, the thermophoretic migration along the temperature gradient separates them spatially to create a light‐directed thermoelectric field pointing from cold to hot. As the particles dispersed in the CTAC solution are positively charged by CTAC adsorption, they are driven by the opto‐thermoelectric field and assembled at the laser spot (hot region).^[^ ^39^ ^]^ Additionally, the CTAC micelles also provide a depletion attraction force for the interparticle bonding, i.e., driven by thermophoresis, the CTAC micelles are depleted from the particle‐to‐particle gap and provide the osmotic pressure to assemble individual particles.^[^ ^40^, ^41^ ^]^ By steering the laser beam, nanoparticles can be dynamically moved and assembled into various nanopatterns.^[^ ^42^, ^43^ ^]^ The trapping process of the nanoparticles under laser illumination is shown in video S1 (Supporting Information). As a first demonstration, we organized 500 nm SiNPs into diverse oligomers, with the particle number ranging from one to ten (Figure 1b). The opto‐thermoelectric field allows to manipulate nanoparticles of different sizes (see Figure S1 (Supporting Information) for the assembly of 300 nm SiNPs) and the reconfigurable geometric control of the assembled nanoclusters (see Figure S2, Supporting Information).

To achieve Fano coupling, we choose BaTiO_3_ nanoparticles (NPs) which exhibit broadband scattering (continuum state), and amorphous silicon particles (SiNPs) of different sizes which show multiple high‐quality‐factor Mie modes (discrete state), as the building blocks. We use in situ dark‐field scattering spectroscopy to identify different nanoparticles and analyze their coupling behavior before and after assembly. More interestingly, the reconfigurable assembly of these hetero‐metamolecules allows real‐time tuning of the cluster structure to manipulate the coupling behavior in the opto‐thermoelectric trap. This versatile optical technique allows to build diverse Fano metamolecules to investigate the underlying coupling physics, which will in turn guide the design of Fano nanostructures with tailorable optical properties.

## 2 Manipulating Fano Heterodimers

**Figure 2:**
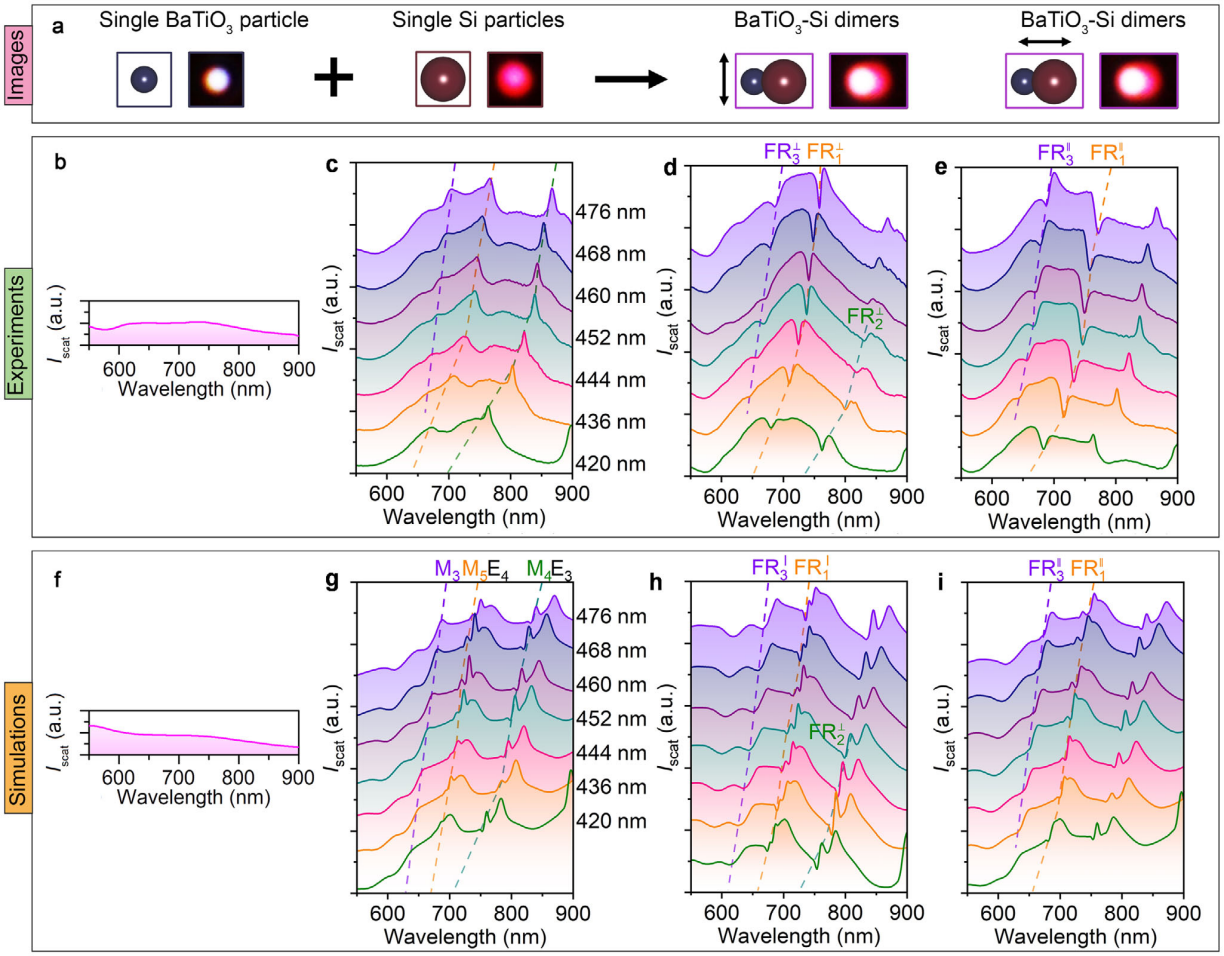
Fano coupling in BaTiO3‐SiNP Heterodimers. A, Scheme and dark‐field optical images of a single BaTiO_3_ NP, a single SiNP, and a BaTiO3‐Si heterodimer (under perpendicular and parallel excitation). b–e, Experimental scattering spectrum of a single BaTiO_3_ NP, single SiNPs with diameters from 420 to 476 nm, BaTiO3‐Si heterodimer at perpendicular polarization, BaTiO3‐Si heterodimer at parallel polarization, respectively. f–i, Simulated scattering spectrum of a single BaTiO_3_ NP, single SiNPs with diameters from 420 to 476 nm, BaTiO3‐Si heterodimers at perpendicular polarization, BaTiO_3_‐Si heterodimers at parallel polarization, respectively. Dashed curves show the evolution of scattering modes in SiNPs and their coupling with the magnetic dipole (M_1_) mode in BaTiO_3_ particles. The electrical (E*_l_*) and magnetic (M*_l_*) spherical Mie modes of l orders are marked in g: l = 1 (dipole), l = 2 (quadrupole), l = 3 (octupole), l = 4 (hexadecapole), etc. ${\mathrm{FR}}_{n}^{⊥}$ and ${\mathrm{FR}}_{n}^{\left|\right|}$ (as well as the dashed curves with the same color) means different sets of Fano resonances at perpendicular polarization and parallel polarization, respectively. The colored rectangle gives the wavelength range of the magnetic dipole mode (M_1_) of individual BaTiO_3_ particles.

**Figure** **2**a shows the scheme and dark‐field optical images of a single 300 nm BaTiO_3_ particle. The single BaTiO_3_ particle appears white under a dark field microscope, matching the broadband scattering spectrum from visible to near‐infrared (Figure 2b). The decomposition of scattering spectra reveals that the broad optical response arises from the magnetic dipole (M_1_) and electric dipole (E_1_) modes (Figure S3, Supporting Information). Unlike BaTiO_3_, Si has a high refractive index (above 3.5) in the visible and near‐infrared regions (300–800 nm), making sub‐micron Si particles excellent Mie resonators. Besides, the spherical shape and smooth surface of the SiNPs improve their quality factor. Figure 2c shows the scattering spectra of individual SiNPs with diameters ranging from 420 to 476 nm. High‐quality‐factor scattering peaks were observed in each particle, with all the modes red‐shift successively when the particle size increases. Scattering intensity below 600 nm is low due to the high optical absorption of amorphous Si, matching the red color in the dark‐field optical image.

When SiNPs and BaTiO_3_ particles formed heterodimers, multiple asymmetric dips appeared in the scattering spectra, indicating Fano coupling. We used polarization‐dependent dark‐field spectroscopy to study the coupling behavior in situ. For a dimer consisting of a 300 nm BaTiO_3_ particle and a 420 nm SiNP, two Fano dips appeared at 680–782 nm when the light polarization was perpendicular to the dimer axis. We studied the spectral evolution by replacing the 420 nm SiNP with SiNPs of different diameters in the heterodimer, as shown in Figure 2c. We can see a clear redshift of the Fano dips when the size of SiNPs increases (${\mathrm{FR}}_{1}^{⊥}$ and ${\mathrm{FR}}_{2}^{⊥}$ indicated by the orange dashed curve and green dashed curve, respectively).

Three spectral features were noted. First, the Fano resonance frequencies were almost the same as the scattering peaks in individual SiNPs. Second, Fano coupling occurred between 650–835 nm (the colored region). New Fano resonances are observed when the high‐quality‐factor scattering peaks of individual SiNPs enter this regime (${\mathrm{FR}}_{3}^{⊥}$ indicated by the violet dashed curve). The multipole decomposition of the scattering spectrum of individual BaTiO_3_ particles (Figure S3, Supporting Information) suggests that the continuum state stems from M_1_ mode instead of E_1_ mode because (i) the Fano line shapes are most distinct at about 750 nm, which coincides with the peak wavelength of M_1_ mode of the BaTiO_3_ particle; (ii) by contrast, no Fano line‐shapes are observed around the peak wavelength (620 nm) of the electric dipole mode of the BaTiO_3_ particle (E_1_ mode). Third, the Fano parameter is tunable by controlling the SiNP size in the heterodimers.

The orientation of the electromagnetic (EM) field in the Mie resonators is sensitive to the polarization of incident light, which significantly modifies the interparticle interaction. When the light polarization is parallel to the dimer axis, ${\mathrm{FR}}_{3}^{\left|\right|}$ exhibits similar spectral evolution as ${\mathrm{FR}}_{3}^{⊥}$. However, distinct coupling behavior is observed at other wavelengths. For instance, ${\mathrm{FR}}_{1}^{\left|\right|}$ shows a similar redshift with the increase of SiNP size, while the Fano parameter is totally different from that of ${\mathrm{FR}}_{1}^{⊥}$. Moreover, ${\mathrm{FR}}_{2}^{\left|\right|}$ is not observed for parallel polarization, where the scattering peaks similar to the ones of individual SiNPs are observed. This observation gives insights into the coupling mechanisms between Si NP and BaTiO_3_ NP resonances under excitation at different polarizations. Apparently, under parallel polarization, the resonances are weakly coupled, which leads to a significant reduction of the Fano contribution to the spectrum, while, under perpendicular polarization, the high‐Q Si NP resonance strongly interacts with the broadband response of the BaTiO_3_ NP, resulting in a pronounced Fano profile. This is evident from the coupled mode theory of the Fano resonances proposed in ref. [15].

To understand the coupling behavior of Fano resonances observed in the heterodimers, we simulated the scattering spectra of individual BaTiO_3_ particle, individual SiNPs, and heterodimers at different light polarizations, respectively, as summarized in Figure 2f–i. The simulated results show similar spectral features to the experimental ones, with additional scattering peaks observed. These additional peaks arise from high‐order Mie modes in the SiNPs, while they are difficult to detect in experiments due to the imperfect spherical shapes. From the simulation of heterodimers, ${\mathrm{FR}}_{2}^{⊥}$ (green dashed curve in Figure 2g) arises from the coupling between M_4_ mode in SiNPs and M_1_ mode in BaTiO_3_ particle at perpendicular polarization. The Fano lineshape disappears when the SiNP size exceeds 460 nm as there is no spectral overlap between these two modes. When the light polarization is parallel to the dimer axis, the Fano coupling did not occur, which is consistent with the experimental observation. At the short wavelength, both ${\mathrm{FR}}_{3}^{⊥}$ and ${\mathrm{FR}}_{3}^{\left|\right|}$ (see the violet dashed curves in Figure 2g,h) arise from the coupling between M_3_ modes in SiNPs and M_1_ modes in BaTiO_3_ particle, which explain the similar spectral evolution at both polarizations. For FR_1_, the polarization‐dependent Fano coupling behavior arises from different mode couplings, i.e., ${\mathrm{FR}}_{1}^{⊥}$ is caused by the coupling between M_5_ mode of SiNPs and M_1_ mode of BaTiO_3_ particle, and ${\mathrm{FR}}_{1}^{\left|\right|}$ is generated by the coupling between E_4_ mode of SiNPs and M_1_ mode of BaTiO_3_ particle.

**Figure 3:**
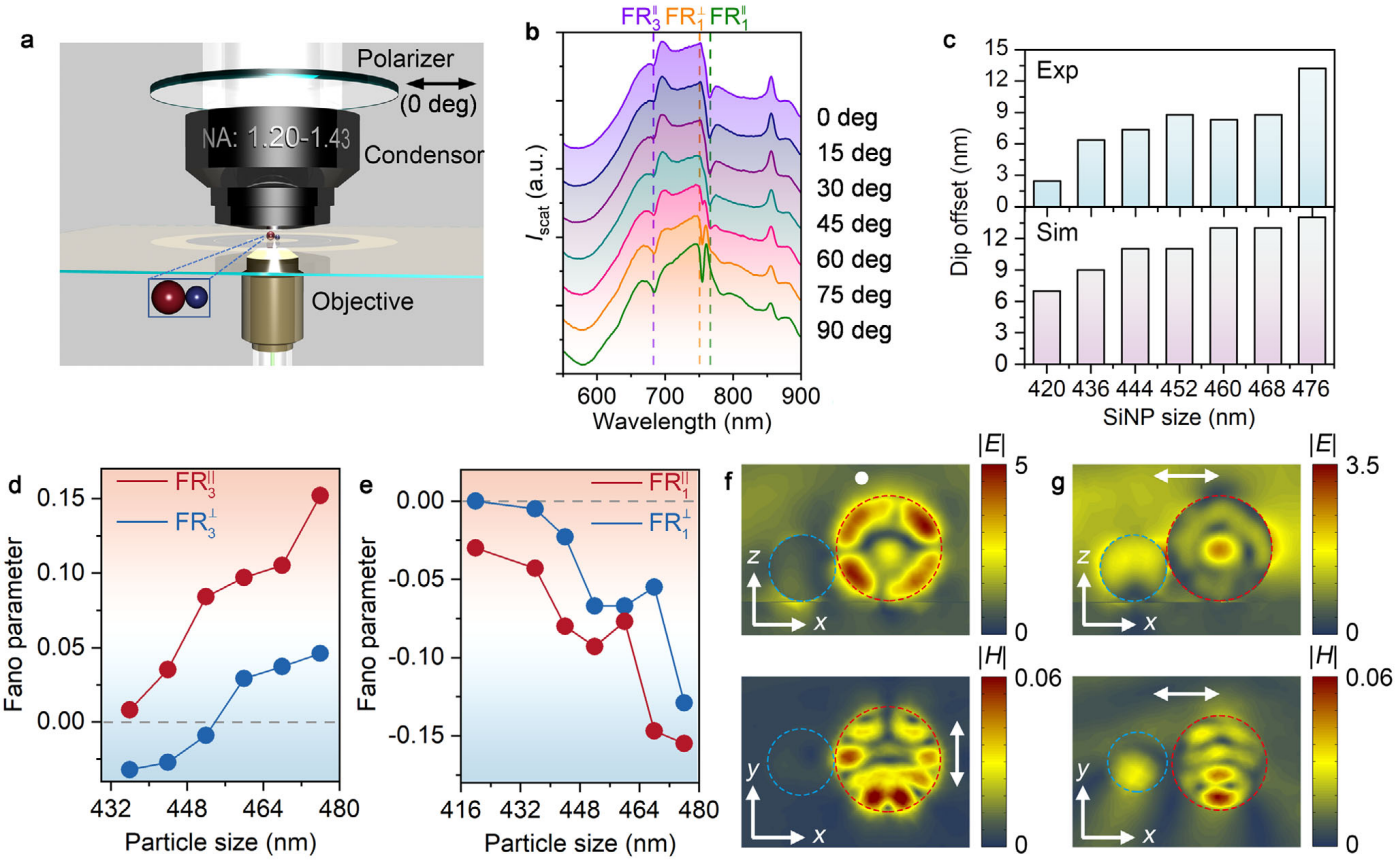
Polarization effects and tunable Fano parameter in 300 nm BaTiO_3_‐500 nm SiNP heterodimers. a, Scheme showing polarization‐dependent dark‐field scattering spectroscopy. b, Polarization‐dependent scattering spectra of 300 nm BaTiO_3_‐500 nm SiNP heterodimers. The violet dashed line represents the evolution of the dip location for FR_3_ when the polarization angle changes from 0 to 90 degrees. The violet, orange, and green dashed lines show the resonance frequencies of FR_3_, ${\mathrm{FR}}_{1}^{⊥}$, and ${\mathrm{FR}}_{1}^{\left|\right|}$, respectively. c, Wavelength offset of Fano resonances FR_1_ between perpendicular and parallel polarizations at different SiNP sizes (experiment, top); Wavelength offset between E_4_ mode and M_5_ mode in single SiNP (simulation, bottom). d, Fano parameter as a function of SiNP size at different polarizations for FR_3_. e, Fano parameter as a function of SiNP size at different polarizations for FR_1_. f, Side view of electric field distribution (top) and top view of magnetic field distribution (bottom) for ${\mathrm{FR}}_{1}^{⊥}$. g, Side view of electric field distribution (top) and top view of magnetic field distribution (bottom) for ${\mathrm{FR}}_{1}^{\left|\right|}$. White arrows indicate light polarization.

To further understand the polarization‐dependent Fano coupling behavior, we measured the dark‐field scattering spectra of the 300 nm BaTiO_3_‐476 nm SiNP heterodimer at different incident light polarizations (see the experimental setup in **Figure** **3**a). As shown in Figure 3b, the dip wavelength of FR_3_ is almost independent on the light polarization, while the Fano parameter changes successively as a function of the polarization angle, indicating similar mode interference at different polarizations. In contrast, FR_1_ exhibits distinct polarization‐dependent behavior. A Fano dip at 754 nm is seen at 90 degrees but becomes inconspicuous and disappears at 0 degrees. Another dip at 765 nm appears and becomes arresting when the polarization degree decreases. At 60 degrees, both Fano dips at 754 and 765 nm coexist, confirming two different mode interferences for FR_1_: M_5_ mode of SiNPs coupling with M_1_ mode of BaTiO_3_ at 90 degrees, and E_4_ mode of SiNPs coupling with M_1_ mode of BaTiO_3_ at 0 degrees. The wavelength offset between the two Fano dips of FR_1_ depends on the SiNP size (see top panel of Figure 3c), explained by the offset between E_4_ and M_5_ modes in single SiNP spectra (see bottom panel of Figure 3c).

To further verify the Fano resonance in the BaTiO_3_‐Si heterodimers, we fit the experimental scattering spectra using an analytical function σ_t_ (ω) = σ_d_ (ω)σ_c_(ω), where the terms of discrete state (σ_d_(ω)) and continuum state (σ_c_(ω)) can be described as^[^ ^44^ ^]^

(1) $$\sigma_{d}\;\left(\omega \right)=\frac{{\left(\frac{\omega^{2}-{\omega_{d}}^{2}}{2\gamma_{d}\omega_{d}}+q\right)}^{2}+b}{{\left(\frac{\omega^{2}-{\omega_{d}}^{2}}{2\gamma_{d}\omega_{d}}\right)}^{2}+1}\;$$

(2) $$\sigma_{c}\;\left(\omega \right)=\frac{a^{2}}{{\left(\frac{\omega^{2}-{\omega_{c}}^{2}}{2\gamma_{c}\omega_{c}}\right)}^{2}+1}\;$$

In these equations, ω_d_ and ω_c_ are the central frequencies, and γ_d_ and γ_c_ are the spectral linewidths of the discrete and continuum states, respectively. *b* is the damping parameter from intrinsic losses, *a* is the maximum resonance amplitude, and *q* is the asymmetric Fano parameter. We note that Equations 1 and 2 can only be effectively applied for approximating the spectral response of the nanostructure within limited spectral ranges, in which the interaction between the discrete and continuum states occurs, while the spectral separation between individual discrete states is sufficiently large. The asymmetric Fano parameter as a function of the SiNP size for ${\mathrm{FR}}_{3}^{⊥}$ and ${\mathrm{FR}}_{3}^{\left|\right|}$ is summarized in Figure 3d. The *q* value of ${\mathrm{FR}}_{3}^{\left|\right|}$ is always positive and it increases monotonously with the SiNP size. In contrast, the *q* value of ${\mathrm{FR}}_{3}^{⊥}$ is switched from negative to positive when the SiNP size exceeds 460 nm. The *q* value of both ${\mathrm{FR}}_{1}^{\left|\right|}$ and ${\mathrm{FR}}_{1}^{⊥}$ decreases with the SiNP size, while a maximal value is observed when the SiNP size is 460 or 468 nm. We examine the EM field distribution of 300 nm BaTiO_3_‐500 nm SiNP heterodimers for FR_1_. At perpendicular polarization, the SiNP shows an EM field of M_5_ mode, with symmetry interrupted by interparticle interference (see Figure S4, Supporting Information). The BaTiO_3_ field is hardly visible due to height differences. At parallel polarization, the electric field on the SiNP surface is observed, which couples with the BaTiO_3_ particle.

The Mie resonance of SiNPs is tunable by controlling their size. Electric and magnetic modes can be tuned to match the magnetic dipole mode of BaTiO_3_ particles for Fano coupling. We assembled heterodimers with SiNPs of 300 and 700 nm in diameter, as shown in Figure S5 (Supporting Information). In 300 nm BaTiO_3_‐300 nm SiNP heterodimers, low‐order electric and magnetic modes appear in the visible and near‐infrared regimes. We observed the coupling between E_2_ mode of the 300 nm SiNPs and M_1_ mode of the BaTiO_3_ particle, leading to Fano resonance at both polarizations. However, we cannot see the Fano interference between the M_2_ mode of the SiNPs and M_1_ mode of the BaTiO_3_ particle, while a suppression of scattering intensity is observed (especially for the perpendicular polarization). The Fano resonance design can also be applied to heterodimers with larger SiNPs, such as 700 nm. As shown in Figure S5 (Supporting Information), the scattering peaks between 650 and 835 nm in single SiNP spectra evolve into Fano dips once they are assembled with BaTiO_3_ particle. However, the scattering peaks of single 700 nm SiNPs in this range stem from high‐order modes, making simulation challenging to explain the coupling behavior.

## 3 Manipulating Fano Heterooligomers

**Figure 4:**
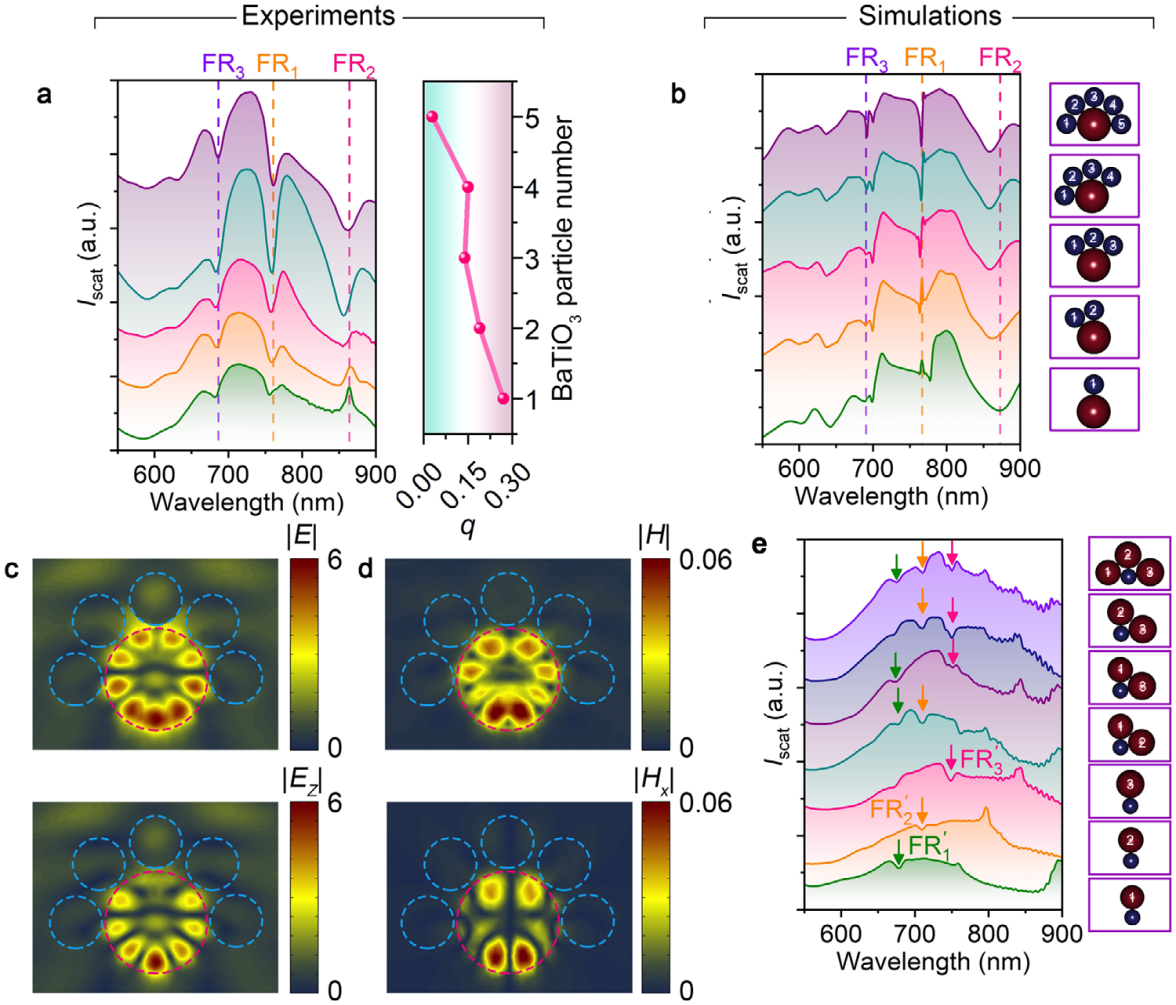
Manipulation of Fano resonances in BaTiO_3_‐SiNP heterooligomers. A, Experimental scattering spectra of heterooligomers with a 500 nm SiNP and varying numbers of BaTiO_3_ particles (bottom to top: one to five, see left panel) and the asymmetric Fano parameter *q* as a function of BaTiO_3_ particles (right panel). b, Simulated scattering spectra of heterooligomers with a 500 nm SiNP and varying numbers of BaTiO_3_ particles. Geometries are shown in the right panels. c, Top view of the electric field distribution (top: |*E*|, bottom: |*E_z_* |) of FR_1_for BaTiO_3_‐SiNP heterooligomers consisting of 5 BaTiO_3_ particles. d, Top view of the magnetic field distribution (top: |*H*|, bottom: |*H_x_* |) of FR_1_for BaTiO_3_‐SiNP heterooligomers consisting of five BaTiO_3_ particles. In c‐d, the diameter of the Si nanoparticle is 500 nm. The resonance wavelength is 760 nm, corresponding to FR_1_ mode marked in panels a and b. e, Experimental scattering spectra of heterooligomers consisting of a 300 nm BaTiO_3_ particle and different numbers of SiNPs (one to three). The arrows with different colors indicate the Fano resonance induced by different SiNPs, i.e., ${\mathrm{FR}}_{1}^{\prime }$ by SiNP_1_, ${\mathrm{FR}}_{2}^{\prime }$ by SiNP_2_, and ${\mathrm{FR}}_{3}^{\prime }$ by SiNP_3_. Geometries are shown in the right panels, where the diameters of SiNP_1_, SiNP_2,_ and SiNP_3_ are 525, 490, and 460 nm, respectively.

Understanding coupling behavior in BaTiO_3_‐SiNP Fano heterodimers guides the design and manipulation of Fano resonance in more complex structures. BaTiO_3_ particles act as continuum states and SiNPs as discrete states, allowing us to tailor Fano resonances by varying their numbers. We expect that adding BaTiO_3_ particles increases the density of continuum states and improves coupling strength. As a proof‐of‐concept, we assembled heterooligomers with a 500 nm SiNP and BaTiO_3_ particles of different numbers (from 1 to 5). The coupling between the SiNP and each BaTiO_3_ particle is similar to each other (Figure S6, Supporting Information). The evolution of scattering spectra verifies our hypothesis, i.e., the Fano dips for both FR_1_ and FR_3_ become deeper when the number of BaTiO_3_ particles increases, suggesting improvement of the coupling strength. For the heterooligomer with five BaTiO_3_ particles, coupling strength is slightly reduced due to the imperfect shape of BaTiO_3_ particles and the structural instability of the metamolecules. Moreover, the addition of BaTiO_3_ particles induces near‐field coupling among the BaTiO_3_ particles and expands the working range of continuum states, providing possibilities to couple with the discrete states beyond 835 nm. In the heterodimer, a scattering peak at 864 nm from the SiNP's M_4_ mode cannot couple with the BaTiO_3_ particle's M_1_ mode due to a lack of spectral overlap. In the heterooligomer with three BaTiO_3_ particles, the peak becomes a Fano dip labeled as FR_2_. Increasing the number of BaTiO_3_ particles further improves coupling strength. Positive and decreasing *q* values are also obtained with the increased number of BaTiO_3_ particles. The observed coupling behavior well matches the simulation (**Figure** **4**b). The EM field distribution of FR_1_from the heterooligomers (Figure 4c,d) further verifies the physical origin of the discrete state, i.e., M_5_ mode in the SiNP.

The distances among the nanoparticles in the oligomers can be modulated by changing the CTAC concentration, which yields different coupling strengths between the BaTiO_3_ and Si nanoparticles. A decreased minimum interaction potential occurs at a shorter bonding length when the CTAC concentration is increased, which increases the osmotic pressure difference and the ionic strength. Thus, the distance can be increased by decreasing the concentration of CTAC (and vice versa). However, the depth of the trapping potential has also to be considered at different interparticle distances. A long distance (low CTAC concentration) corresponds to a shallow trapping potential and a low robustness of the assembly, while a short distance (high CTAC concentration) corresponds to a deep trapping potential. In addition, at a higher CTAC concentration, the manipulation of the nanoparticle becomes challenging because the depletion attraction force between the nanoparticles and the substrate is increased. Thus, in our work, we chose a moderate CTAC concentration of 5 mM for the assembly.^[^ ^43^ ^]^

Besides the engineering of continuum states in the heterooligomers, we anticipate that the selection and addition of SiNPs into the heterooligomers provide another degree of freedom to tailor the Fano resonance. For verification, we picked up a BaTiO_3_ particle and three SiNPs with different sizes (labeled as SiNP_1_, SiNP_2_, and SiNP_3_), with the single‐particle scattering spectra summarized in Figure S7 (Supporting Information). First, we assembled each SiNP with the BaTiO_3_ particle to form three different heterodimers and recorded their scattering spectra, respectively. The Fano resonance arising from the coupling between M_5_ mode of the SiNP and M_1_ mode of the BaTiO_3_ particle in these heterodimers is written as ${\mathrm{FR}}_{1}^{\prime }$, ${\mathrm{FR}}_{2}^{\prime }$, and ${\mathrm{FR}}_{3}^{\prime }$, respectively. Then, we assembled the BaTiO_3_ particle with two of the SiNPs to form three types of heterotrimers. Interestingly, we can see clearly an add‐up effect. That means, when SiNP*_i_* and SiNP*_j_* (*i*, *j* = 1, 2, or 3) are assembled with the BaTiO_3_ particle to form a trimer, the Fano resonance in both heterodimers (${\mathrm{FR}}_{i}^{\prime }$ and ${\mathrm{FR}}_{j}^{\prime }$) can be observed in the trimer. Moreover, we assembled the four particles together to form a heterotetramer and observed that ${\mathrm{FR}}_{1}^{\prime }$, ${\mathrm{FR}}_{2}^{\prime }$, and ${\mathrm{FR}}_{3}^{\prime }$ co‐exist in the scattering spectrum. This add‐up effect provides opportunities to design versatile and multiple Fano resonance.

## 4 Conclusion

Using a light‐directed thermoelectric field, we demonstrated the dynamic manipulation of Fano coupling in an optical trap. Dielectric nanoresonators with distinct optical properties are trapped and assembled into heterodimers or heterooligomers to study Fano interference. Unlike the Fano theory based on mode hybridization, we found that individual dielectric particles support continuum or discrete states, allowing precise design and control of Fano interference through selective assembly. Using SiNPs (discrete states) and BaTiO_3_ particles (continuum states) as examples, we successfully tuned the Fano coupling by controlling particle size, composition, and light polarization. By tuning the size of the SiNPs in the SiNP‐BaTiO_3_ dimer, we have demonstrated the capability of our method to precisely control the Fano parameter and, by that means, achieve the desired spectral lineshape and resonance detuning, which is necessary for Fano‐based optical sensors and nanolasers. Moreover, we observe an interesting add‐up effect in the BaTiO_3_‐SiNP heterooligomers, which allows to design of multiple Fano resonances in a versatile manner. The on‐demand manipulation of Fano resonance in this work provides opportunities to gain insight into the Fano coupling theory, which will find a lot of applications in active photonics and bio‐sensing.

## 5 Experimental Section

### Sample Preparation

SiNPs were synthesized following the protocols in previous works.^[^ ^45^ ^]^ Both trisilane and n‐hexane were added to a titanium reactor placed in a nitrogen glove box. The reactor was then sealed and taken out from the glove box. For the decomposition of the trisilane, the reactor was heated to the target temperature for 10 min. The reactor was then cooled down to room temperature in an ice bath. The SiNPs were then washed with chloroform by centrifuging the samples at 8000 rpm for 5 min. 300 nm BaTiO_3_ NPs in this work were commercially purchased from Nanostructured & Amorphous Materials, Inc. The gold film was prepared by deposition of 4 nm gold on a SiO_2_ coverslip through thermal deposition and followed by annealing at 550 °C for 2 h.

### Optical Assembly and Measurement

A 532 nm continuous‐wave laser (Coherent) was used for the opto‐thermoelectric assembly. The laser beam was expanded using a 5× beam expander and directed to the Nikon Ti‐E microscope. A 100× oil objective (numerical aperture: 0.5–1.3) was used to focus the laser beam on the gold film. BaTiO_3_ NPs and SiNPs were dispersed in 5 mm CTAC solution and confined by a spacer on the gold film. The focused laser beam (0.2 mw) heats the substrate and creates the thermoelectric field which captures BaTiO_3_ NPs and SiNPs for assembly. For the measurement of dark‐field scattering spectra, an oil‐immersed condenser (NA:1.20–1.43) was used to focus the white light on the gold film, while the polarization was tuned by a linear polarizer (LPNIRE100‐B, Thorlabs Inc.). The dark‐field scattering signal was collected and directed to an Andor spectrometer for in situ measurement.

## Conflict of Interest

The authors declare no conflict of interest.

## Supporting information



Supporting Information

Supplemental Video 1

## Data Availability

The data that support the findings of this study are available from the corresponding author upon reasonable request.
