# Supplementary material for: Manipulating Fano Coupling in an Opto‐Thermoelectric Field
Source: Adv Sci (Weinh). 2025 Jan 21;12(10):2412454. doi: 10.1002/advs.202412454 (PMC11904968; doi:10.1002/advs.202412454)
Supplement: Supplementary file 1 — Supporting Information [file ADVS-12-2412454-s001.docx]

**Supplementary Information**

**Manipulating Fano coupling in an opto-thermoelectric field**

Linhan Lin^1,†,*^, Sergey Lepeshov^2,†^, Alex Krasnok^3,*^, Yu Huang^4^, Taizhi Jiang^5^, Xiaolei Peng^6^, Brian A. Korgel^5,6^, Andrea Alù^7,8,9^, and Yuebing Zheng^6,10,*^

^1^State Key Laboratory of Precision Measurement Technology and Instruments, Department of Precision Instrument, Tsinghua University, Beijing 100084, People’s Republic of China

^2^Department of Electrical and Photonics Engineering, DTU Electro, Technical University of Denmark, Building 343, DK-2800 Kgs. Lyngby, Denmark

^3^Department of Electrical and Computer Engineering, Florida International University, Miami, Florida 33174, USA

^4^School of Physics and Electronics, Hunan University, Changsha 410082, China

^5^Mc Ketta Department of Chemical Engineering, The University of Texas at Austin, Austin, TX 78712, USA

^6^Materials Science & Engineering Program and Texas Materials Institute, The University of Texas at Austin, Austin, TX 78712, USA

^7^Department of Electrical and Computer Engineering, The University of Texas at Austin, Austin, TX 78712, USA

^8^Photonics Initiative, Advanced Science Research Center, City University of New York, New York, NY 10031, USA

^9^Physics Program, Graduate Center, City University of New York, NY 10016, USA

^10^Walker Department of Mechanical Engineering, The University of Texas at Austin, Austin, TX 78712, USA

*E-mail: linlh2019@tsinghua.edu.cn; akrasnok@fiu.edu; zheng@austin.utexas.edu

^†^These authors contributed equally to this work.


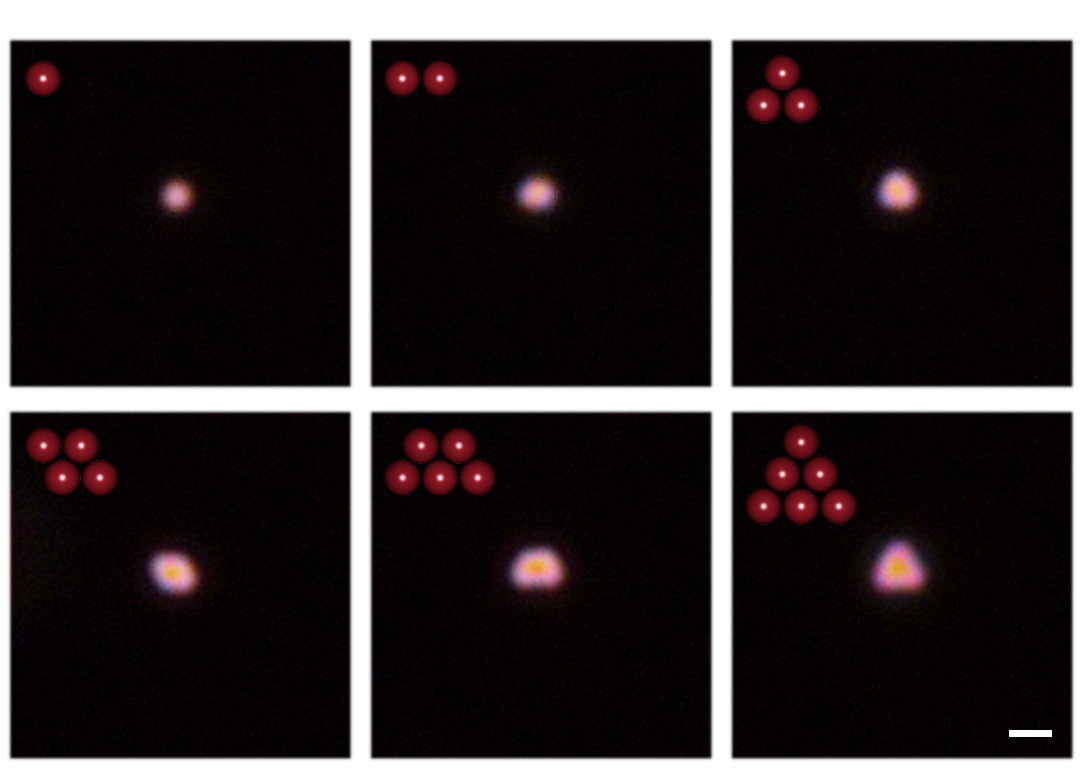


**Figure S1. Dark-field optical images showing assembly of 300 nm SiNPs using in an opto-thermoelectric field.** Inset: Schemes of the assemblies. Scale bar: 1 μm.


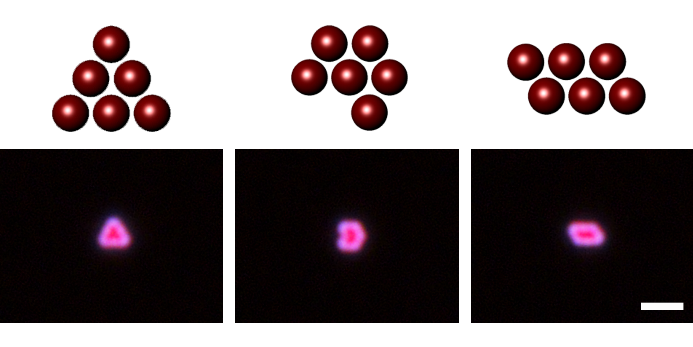


**Figure S2. Reconfigurable assembly of six 500 nm SiNPs. Top: Schemes of the assemblies. Bottom: Dark-field optical images of the assemblies.** Scale bar: 2 μm.


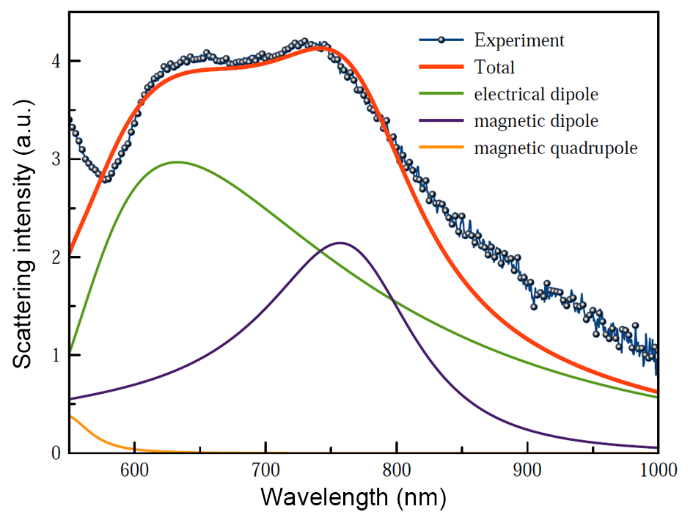


**Figure S3. Multipole decomposition of the scattering spectrum of individual BaTiO_3_ particle.**


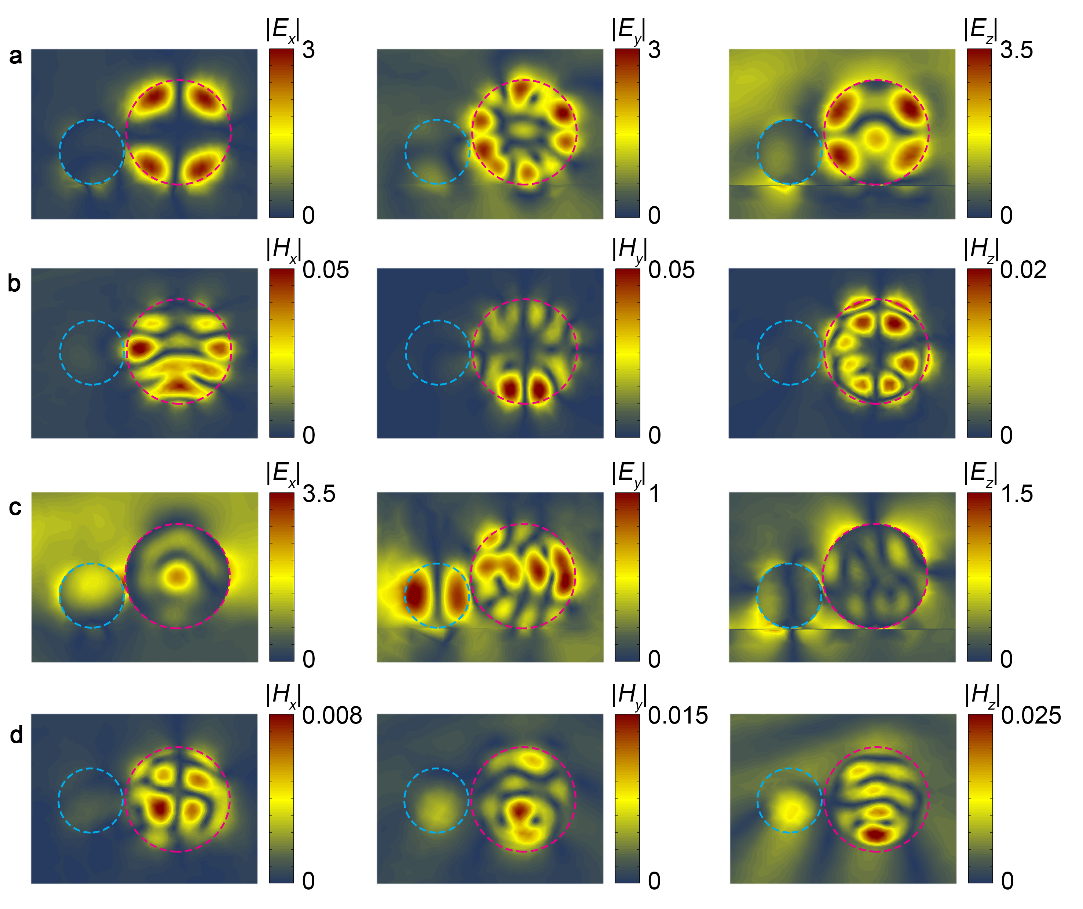


**Figure S4. Electromagnetic field distribution in the 300 nm BaTiO_3_-500 nm SiNP heterodimers.** **a**-**b**, Side view of electric field distribution (a) and top view of magnetic field distribution (b) for $\mathrm{FR}_{1}^{\perp}$. **c**-**d**, Side view of electric field distribution (d) and top view of magnetic field distribution (d) for $\mathrm{FR}_{1}^{||}$.


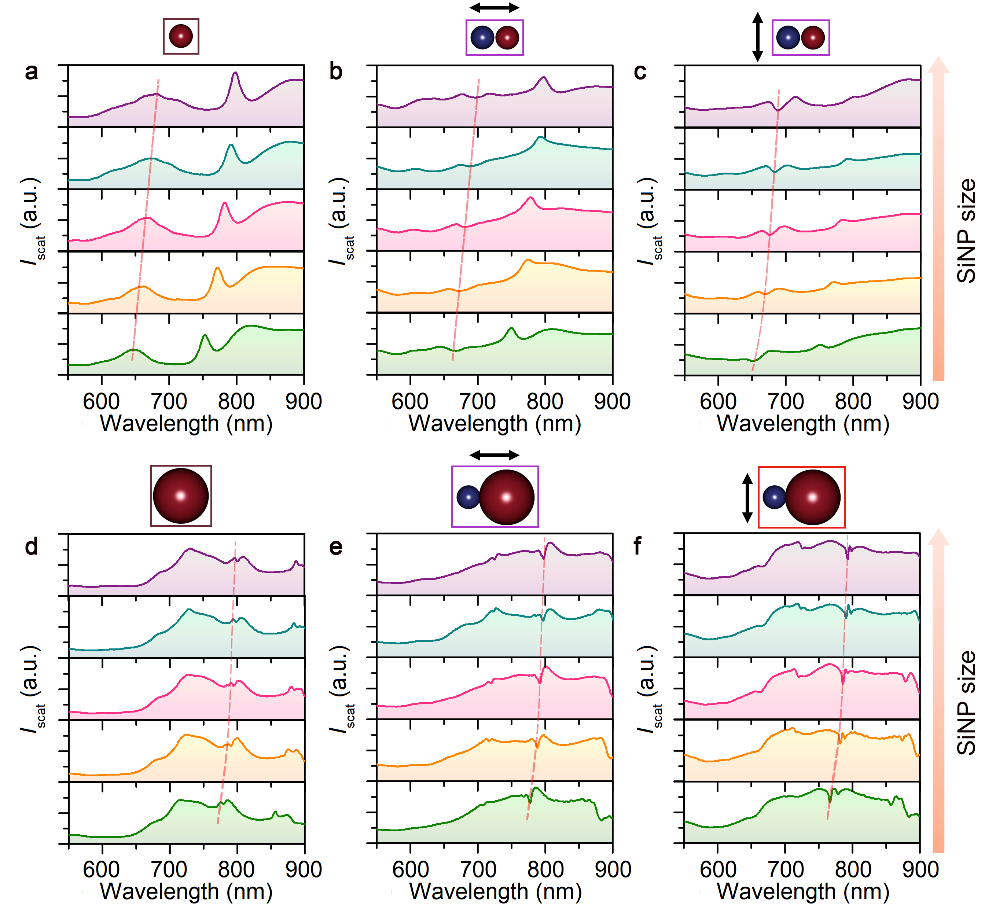


**Figure S5 Manipulating Fano resonance in 300 nm BaTiO_3_-300 nm SiNP heterodimers and 300 nm BaTiO_3_-700 nm SiNP heterodimers. a,** Scattering spectra of individual 300 nm SiNPs. **b,** Scattering spectra of 300 nm BaTiO_3_-300 nm SiNP heterodimers with polarization parallel to the dimer axis. **c,** Scattering spectra of 300 nm BaTiO_3_-300 nm SiNP heterodimers with polarization perpendicular to the dimer axis. **d,** Scattering spectra of individual 700 nm SiNPs. **e,** Scattering spectra of 300 nm BaTiO_3_-700 nm SiNP heterodimers with polarization parallel to the dimer axis. **f,** Scattering spectra of 300 nm BaTiO_3_-700 nm SiNP heterodimers with polarization perpendicular to the dimer axis.


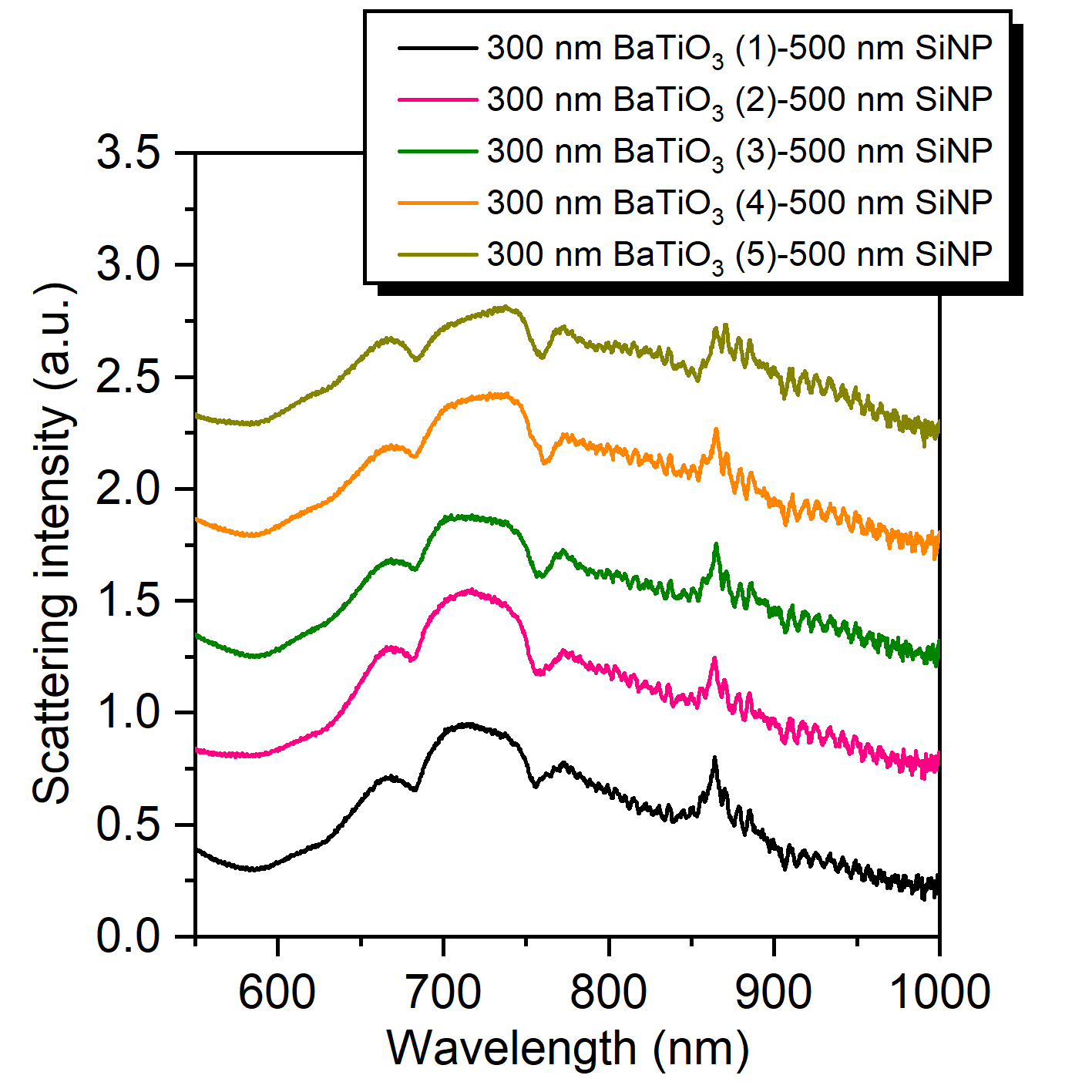


**Figure S6. Scattering spectra of 300 nm BaTiO_3_-500 nm SiNP heterodimers.** These heterodimers are composed of the same 500 nm SiNP and different BaTiO3 nanoparticles while exhibiting similar spectral features.


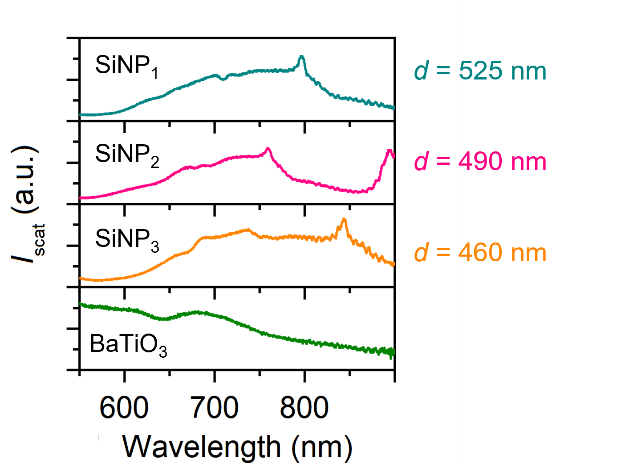


**Figure S7. Scattering spectra of individual nanoparticles in the heterooligomers consisting of a 300 nm BaTiO_3_** **and different numbers of SiNPs.** The diameters of SiNP_1_, SiNP_2_ and SiNP_3_ are 525 nm, 490 nm and 460 nm, respectively.
